# *Amazonopsis*, an unusual new genus of riffle beetle from South America with two new species (Coleoptera, Elmidae, Elminae)

**DOI:** 10.3897/zookeys.803.28124

**Published:** 2018-12-06

**Authors:** Cheryl B. Barr

**Affiliations:** 1 Essig Museum of Entomology, 1101 Valley Life Sciences Bldg., University of California, Berkeley, California 94720, USA University of California Berkeley United States of America

**Keywords:** Aquatic beetles, French Guiana, Neotropics, peri-Amazonian, Peru, sexual dimorphism, taxonomy, Venezuela

## Abstract

*Amazonopsis***gen. n.** is described to include *A.theranyi***sp. n.** from Peru, Venezuela and French Guiana, and *A.camachoi***sp. n.** from Venezuela. The descriptions are accompanied by figures illustrating the male and female habitus of *A.theranyi*, the male habitus of *A.camachoi*, and male genitalia of both species. *Amazonopsistheranyi* exhibits pronounced secondary sexual dimorphism which likewise may be a characteristic of the genus. *Amazonopsis* males have modified protarsal and mesotarsal claws, a pair of small spines on the anterior prosternum, and a pair of ventrally directed processes on the posterior metaventrite. Females of *A.theranyi* display a pair of unique, oval perforations in the cuticle of the pronotum and have unmodified claws; females of *A.camachoi* are unknown. Descriptions are furnished of the stream habitats and microhabitats where the study specimens were collected.

## Introduction

Presently 40 genera and over 250 species of the aquatic beetle family Elmidae are known from South America (Manzo 2005, Maier and Spangler 2011, Maier 2012), including 25 genera and 102 species from the Amazon region (Passos et al. 2010). The Guiana Shield in northern South America so far has yielded 22 genera including the endemics *Elachistelmis* Maier, 2012, *Hypsilara* Maier & Spangler, 2011, *Jolyelmis* Spangler & Faitoute, 1991, *Neblinagena* Spangler, 1985, *Neolimnius* Hinton, 1939, and *Roraima* Kodada & Jäch, 1999. In recent years, taxonomic checklists have been compiled for several South American countries, including Argentina (Manzo and Archangelsky 2014), French Guiana (Queney 2012), Ecuador (Monte and Mascagni 2012), Paraguay (Shepard and Aguilar Julio 2010), Peru (Shepard and Chaboo 2015), and parts of Colombia (González-Córdoba et al. 2015, 2016) and Brazil (Fernandes 2010, Passos et al. 2009), and for the Neotropical Region in general (Segura et al. 2013). Current research activity on the family in South America results in frequent articles describing new taxa.

Specimens of an unusual new genus and species of elmid were collected in the Amazon Basin of southeastern Peru in 2012 during a water quality survey of stream macroinvertebrates conducted by the Stroud Water Research Center (Avondale, Pennsylvania, USA) in conjunction with the Amazon Center for Environmental Education and Research (ACEER) (West Chester, Pennsylvania, USA) (Howard 2012, Sweeney 2014). In 2016, I collected additional specimens of the genus in French Guiana while participating in the Diversity of Aquatic Insects of French Guiana (DIAG) project (Clavier 2017). Two specimens from Venezuela, collected in 1985 and 2004, were found in different museum collections. The larva is unknown.

The purpose of this paper is to describe this distinctive new genus of elmid, its two new species, and the stream habitats from which they were collected.

## Materials and methods

Four specimens collected by the Stroud–ACEER project in Peru were taken from artificial leaf packs, consisting of plastic mesh bags filled with 7 g of fresh *Ingaedulis* Martius (Fabaceae) leaves, which were deployed in the stream for about four weeks (Howard 2012, Sweeney 2014). After retrieval of the leaf packs, the contents were examined for specimens which were preserved in 90% ethanol. The specimens were then taxonomically sorted and distributed to specialists for identification. I was involved with identification of the Elmidae collected during the project. Subsequently, while a member of the DIAG team in French Guiana, I collected two specimens from a stream in the interior. They were dislodged into the current from naturally occurring leaf and stick packs and captured in a D-frame aquatic net. They were then preserved in 95% ethanol in the field. Two specimens from Venezuela were borrowed from the National Museum of Natural History (Washington, DC, USA) and the Museo del Instituto de Zoologia Agrícola (Maracay, Venezuela).

Water quality measurements of the streams were provided by the Stroud Water Research Center for the specimens collected in Peru during the ACEER project (Sweeney 2014), and by Hydreco Guyane (Kourou, French Guiana) for those collected in French Guiana during the DIAG project (S. Clavier in litt.). Those from Cerro de la Neblina, Venezuela, were obtained from a publication describing species of *Stegoelmis* (Elmidae) collected concurrently at the site (Spangler 1990).

Specimens were examined in the lab using a Leica MZ 12.5 stereo microscope fitted with an ocular micrometer. Measurements of total body length represent the length of the pronotum plus the length of the elytra, excluding the head and the variable space between the pronotum and elytra; measurements of body width are composed of both elytra at their widest point. The habitus images were taken with a Visionary Digital BK Plus Lab System fitted with a Canon EOS 7D camera. A Syncroscopy AutoMontage system was used for the genitalia images. All of the specimens are double-mounted on card points and pins. Males have the aedeagi removed and stored with glycerin in genitalia vials beneath the donor specimens.

Label data are reported verbatim as found on the specimen labels: “ / ” indicates separate lines on one label and “ // ” indicates separate labels. Brackets “ [] ” indicate additional clarifying information not included on the label.

The distribution map is an alteration of a map of South America provided free on the internet by d-maps (2018) of Trets, France.

Specimens will be deposited in the following institutions:

**EMEC** Zoologische Staatsammlung München, Munich, Germany

**MALUZ** La Universidad del Zulia, Maracaibo, Venezuela

**MNHN** Muséum National d’Histoire Naturelle, Paris, France

**MUSM** Museo de Historia Natural, Universidad Nacional Mayor de San Marcos, Lima, Peru

**USNM** National Museum of Natural History, Washington, DC, USA

## Results

### Amazonopsis gen. n.

Taxon classificationAnimaliaColeopteraElmidae

http://zoobank.org/D193A59E-A5B8-4A0C-BC42-E82454C15902

Figs 1 , 2 , 3 , 4 , 5 , 6 , 7, 8 , 9 , 10

#### Type species.

*Amazonopsistheranyi* sp. n.

#### Other species.

*Amazonopsiscamachoi* sp. n.

#### Diagnosis.

**Figure 1.**
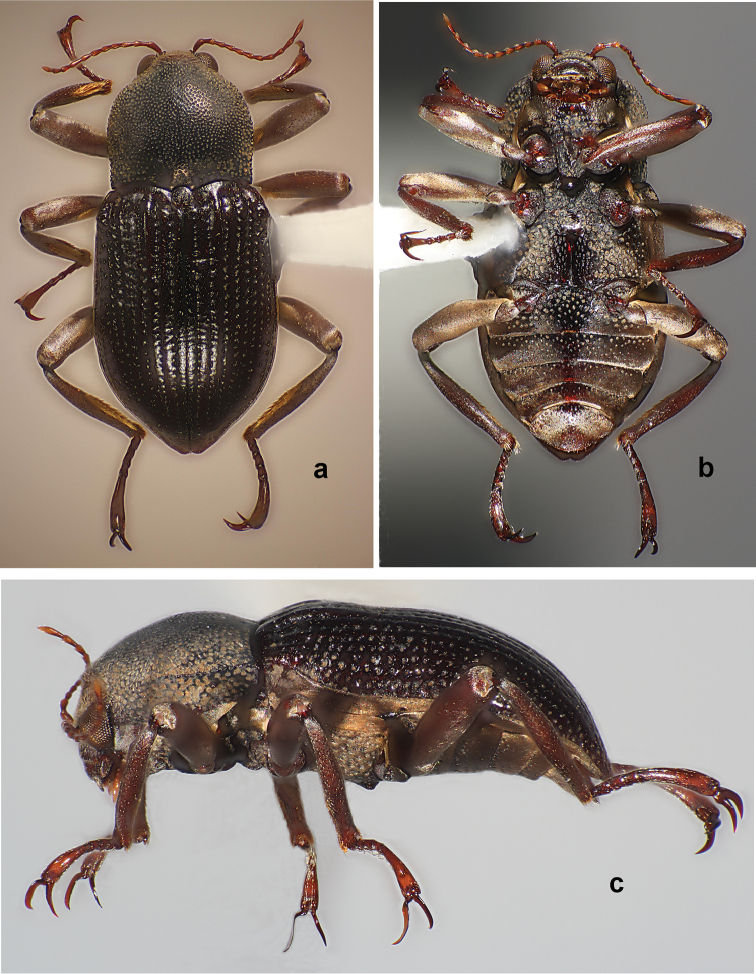
*Amazonopsistheranyi* sp. n., paratype male from Peru, length 4.15 mm **a** Dorsal habitus **b** Ventral habitus **c** Lateral habitus.

The flattened and bent pro- and mesotarsal claws of the males (Figs 1, 4, 9) and the pronotal perforations of the females (Fig. 3) are unique among the Elmidae, and both sexes lack pronotal and elytral carinae.

#### Generic description.

**Male.** Body stout, elongate-oval, at least 2× as long as wide; convex dorsally. Surface of dorsum and parts of venter covered by thin, pale gray, microreticulate plastron; thick, glossy plastron present laterally on sterna and adjacent surfaces of coxae, legs (except tarsi), lower margin of hypomeron, abdominal ventrites (except along midline), and entire epipleuron; head (vertex, frons, clypeus) and pronotum with short, broad, flat, pale yellow setae. Tibial cleaning fringes well-developed, formula 2-2-1. *Head.* Antenna filiform, with 11 antennomeres. Vertex with V-shaped carina opening anteriorly; frons slightly elevated between eyes; eyes large, subcircular in outline. Clypeus rectangular, wider than long. Labrum rectangular, not as wide as clypeus. Mandible with three short, rounded, apical teeth. Maxillary palpus with four palpomeres. Labial palpus with three palpomeres. *Pronotum.* Subquadrate, slightly wider than long, widest at midlength; without carinae or gibbosities. Disc, including punctures, covered with pale microreticulate plastron. Scutellum subcircular to ovate, flat. *Elytron.* Elongate, about 3× as long as wide; without carinae except for swollen, raised base of third interval; humeral angles protuberant. Disc with 10 longitudinal rows of coarse, deep punctures; row 10 near margin with much smaller punctures than rows 1–9. Epipleuron with excavation adjacent to marginal lobe of abdominal ventrite 4. Surface of disc, including punctures, with thin, pale microreticulate plastron, often abraded; cuticle beneath very shiny, reddish-brown. *Leg.* Femur and tibia covered with thin, shiny layer of dense plastron; tarsus without plastron. Prothoracic leg shortest, metathoracic leg longest. Pro- and mesocoxa globose, metacoxa transverse. Pro- and mesotibia each with anterior and posterior cleaning fringes of long setae; metatibia with a single, posterior fringe. Claws long, without basal teeth; protarsal and mesotarsal inner and outer claws dissimilar; protarsal inner and mesotarsal outer claws enlarged, laterally flattened, bent at base; protarsal outer and mesotarsal inner claws smaller, narrower; metatarsal claws shorter, flattened, but basically unmodified. *Venter.* Pale microreticulate plastron present on ventral surfaces except at midline; plastron yellow and most evident near lateral thoracic margins and on abdominal ventrites. Prosternum slightly shorter than metaventrite; anterior margin curved posteriad, bounded by pair of small, ventrally directed spines; prosternal process about 2× as long as wide, margins raised, bluntly rounded at apex. Mesoventrite shortest; deep cavity present to accommodate prosternal process. Metaventrite longest, slightly longer than prosternum; metathoracic discrimen distinct; posteromedial margin with pair of ventrally directed processes. Abdomen with five ventrites; ventrites 1–4 decreasing in length posteriorly, ventrite 5 longer than ventrite 1; ventrites narrower medially and wider laterally; ventrite 4 lateral margin with lobe to link with groove on epipleuron, posterior margin strongly raised and rounded. *Genitalia.* Trilobate, typical form.

**Female.** Although the female of *A.camachoi* is unknown, it is possible, if not likely, that secondary sexual dimorphism is a generic characteristic. Females of *A.theranyi* exhibit the following differences (Fig. 3): pronotum with two, moderately large, oval perforations of the cuticle on either side of the midline; claws all unmodified; prosternum and metaventrite without paired, ventrally directed spines or processes.

#### Etymology.

“Amazon”, a Greek word for a legendary race of warrior women, refers to the robust, unique features of the beetles as well as the provenance of the genus; plus “-opsis” from the Greek meaning “look, appearance, likeness.” Gender, feminine.

#### Comparative notes.

In Manzo (2005), *Amazonopsis* keys to the couplet containing *Pagelmis* Spangler, 1981 and *Stenhelmoides* Grouvelle, 1908, because of its extensive dorsal plastron and lack of pronotal and elytral carinae, but matches neither genus. *Amazonopsis* has a generally uniform distribution of plastron (except for abraded areas), while *Pagelmis* (Spangler 1981) and *Stenhelmoides* (Grouvelle 1908) exhibit characteristic patterns. In addition, the epipleuron-clasping projection on abdominal ventrite 4 in *Amazonopsis* is lacking in *Pagelmis* and *Stenhelmoides*. *Amazonopsis* is similar to *Portelmis* Sanderson, 1953, in that both lack elytral carinae, possess a V-shaped ridge behind the eyes, and have a pair of abdominal lobes linking the abdomen to the elytra (Sanderson 1953). Unlike *Amazonopsis*, *Portelmis* has the lateral margin of the fifth, rather than the fourth, abdominal ventrite prolonged to link with the elytral epipleuron; most species have pronotal sublateral carinae; and the tibial cleaning fringes are poorly developed. *Xenelmis* Hinton, 1936, has a similar body form, but at less than 2 mm long is less than half the size of *Amazonopsis*. Like *Amazonopsis*, *Xenelmis* lacks pronotal carinae, but dissimilarly has two sublateral elytral carinae (Hinton 1936).

### Amazonopsis theranyi sp. n.

Taxon classificationAnimaliaColeopteraElmidae

http://zoobank.org/ED249675-189F-484F-8E9C-2A42FCA6B81B

Figs 1 , 2 , 3 , 4 , 5 , 6 , 7–8

#### Type material.

**Holotype male** deposited in MUSM, labeled: “**PERU**: [Dpto.] Madre de Dios / Tambopata, Quebrada / Santo Rosario, el. 230 m / −12.8788, −69.7396 / 29-V-2012, T. Gonzales // collected from artificial / leaf pack of *Ingaedulis* / leaves, ACEER-Stroud / project 2012–2013 // HOLOTYPE / *Amazonopsis* / *theranyi* Barr” [red label, handwritten]. **Allotype female** deposited in MUSM, labeled “PERU: [Dpto.] Madre de Dios / Tambopata, Quebrada / Santo Rosario, el. 230 m / −12.8788, −69.7396 / 4-VII-2012, T. Gonzales // collected from artificial / leaf pack of *Ingaedulis* / leaves, ACEER-Stroud / project 2012–2013 // ALLOTYPE / *Amazonopsis* / *theranyi* Barr” [red label, handwritten]. **Paratypes** (2) locality as above, 29-V-2012 [yellow labels, printed] (1 M & 1 F, EMEC).

#### Additional material examined.

**french guiana** / ca. 4 km ESE Saül / Cr.[ique] Nouvelle France / 03.6063, −53.1762 / 9-XI-2016, C. B. Barr // Parc Amazonien / de Guyane just / below Point Chaud / coll. from leaf pack (1 F, EMEC); as above, ca. 4.5 km SE Saül / Cr.[ique] Nouvelle France / 03.5972, −53.1779 / 9-XI-2016, C. B. Barr // Parc Amazonien / de Guyane at / Courant Doublé / coll. from leaf pack (1 F, MNHN); **VENEZUELA**. T. F. Amaz.[onas] / Cerro de la Neblina / 1 km S Basecamp / 0°50'N, 66°10'W / 140 m, 8 Feb. 1985 // Small pool full of / dead leaves; rain- / forest ridge / W. E. Steiner & R. Halling collrs. (1 M, USNM).

#### Diagnosis.

**Figure 2.**
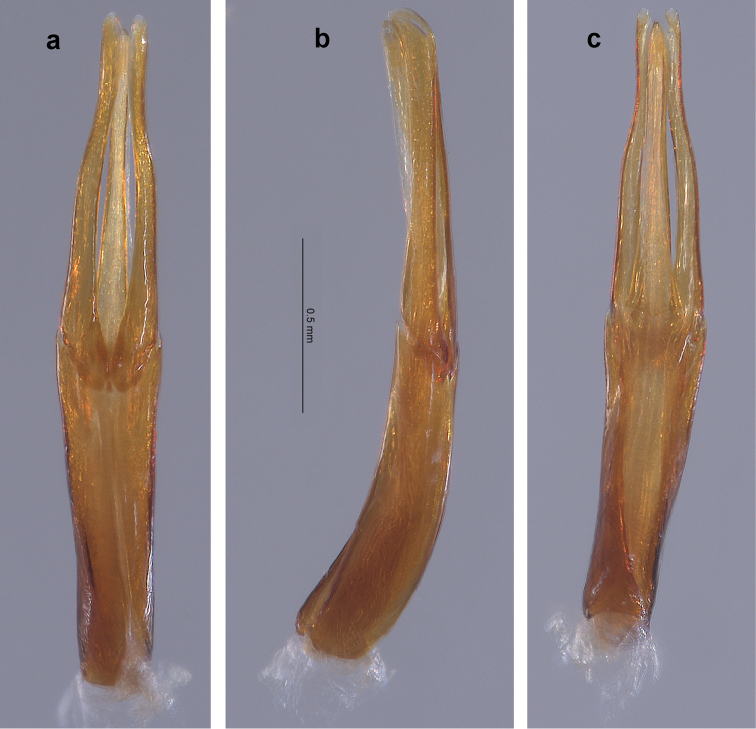
*Amazonopsistheranyi* sp. n., male genitalia of holotype from Peru **a** Dorsal view **b** Lateral view **c** Ventral view.

*Amazonopsistheranyi* males (Figs 1, 2, 4, 5) differ from those of *A.camachoi* (in parentheses) (Figs 9, 10) by the following characters: elytra with low protuberances at humeral angles (protuberances prominent), third elytral interval slightly raised (prominently raised), all intervals with fine, sparse setae (odd-numbered intervals with longitudinal rows of thick setae); protarsomere 5 with sparse setae barely extending to base of claws (two apical clusters of long, curved setae extending well beyond base of claws); protarsal claws elongate, moderately curved (short, strongly curved), outer claw without inner tooth (with inner tooth), claws of similar width (outer claw narrower); outer mesotarsal claw shorter than tarsomere 5 (as long as tarsomere 5) and 2× wider than inner claw (at least 3× wider); distance between prosternal spines narrower than labrum (as wide as); metaventrite with pair of small, tooth-like processes (prominent, lobe-like); male genitalia with penis as wide at base as paramere base (much wider than), penis narrower at midlength than paramere at midlength (wider than), phallobase length subequal to that of parameres (longer than).

#### Description.

**Holotype male.** Length, 4.20 mm; width, 1.90 mm. Cuticle mostly covered with pale, thin microreticulate layer dorsally and ventrally, with thicker, glossy, golden-yellow plastron ventrolaterally on thoracic sterna and abdominal ventrites; cuticle shiny, dark reddish-brown where exposed. *Antenna.* Yellow-brown. Antennomeres 1–10 clavate, antennomere 11 fusiform; antennomeres 1 and 2 each stouter than 3–10 which are of similar size and shape; antennomeres 3–10 each with dense tuft of setae at apicoventral margin, overlapping base of next; antennomere 11 with an elongated patch of short setae near the ventral apex. *Head.* Vertex, frons and clypeus covered with pale, microreticulate plastron and broad, flat, yellow setae. Clypeus dark brown, barely emarginate at center of apical margin, setae slightly less dense than on vertex and frons. Labrum dark brown, barely emarginate, apicolateral angles broadly rounded; surface with small, evenly spaced punctures and short, fine setae; apical and lateral margins with fringe of pale, dense setae, longest laterally. Mandible with three short, rounded, apical teeth. Maxillary palpus yellow-brown; palpomere 4 slightly flattened and curved, longer than 1–3 combined, with oval patch of sensillae at apex. Labial palpus with palpomeres 1 and 2 short, dark brown; palpomere 3 longer than 1 and 2 combined, yellow-brown, ovoid and moderately flattened. *Pronotum.* Length, 1.20 mm; width, 1.50 mm. In dorsal view, lateral margins coarsely granulate, unevenly arcuate; anterior margin trisinuate, strongly arcuate at middle; anterolateral angles acute, depressed. In lateral view, moderately convex. Disc covered with pale microreticulate plastron and deep, closely spaced, coarse punctures; punctures larger towards the lateral margins, smaller towards the midline; punctures generally spaced a diameter apart; punctures lined with plastron and associated with very short, erect setae; anteromedial disc and lateral areas with broad, flat, recumbent, yellow setae. Center of midline with narrow, lightly impressed, bare, longitudinal line; length about ½ that of pronotum. Scutellum subcircular. *Elytron.* Length, 3.00 mm; width, 0.95 mm. Surface covered with pale, thin microreticulate plastron, abraded at center of disc; punctures striate, deep, coarse, lined with plastron; intervals of striae with fine, sparse setae. Humeral angle with low, knob-like protuberance; base of third interval slightly swollen and raised; lateral margins smooth, recurved with narrow, longitudinal band of hypomeron plastron visible; shallow sulcus about one interval wide adjacent to lateral margin, extending from humeral angle to apical 1/5; elytra constricted at apical 1/5 at point of linkage with abdominal ventrite 4 lateral lobe; apex evenly rounded, moderately produced. *Legs.* Femora and tibiae covered by thin layer of shiny, pale yellow plastron, sparsely setose and shallowly punctate; tarsus red-brown, without plastron. Procoxa posterior surface coarsely punctate; dense patch of long, golden-yellow setae present on lateral face. Prothoracic leg with tibia longer than femur, tarsus shorter; profemur with oval patch of long, recumbent, golden-yellow setae on anterior surface near base; protibia with pair of cleaning fringes nearly ½ as long as tibia, posterior fringe slightly shorter; protarsus with tarsomeres 1–4 bearing dense tufts of moderately long setae in two rows at apicoventral margins; tarsomere 5 longer than the others combined, with moderately long setae on ventral surface and a few longer, golden-yellow setae at apex which barely extend over base of claws. Protarsal claws dissimilarly shaped, long, laterally flattened, sharply acute; inner claw enlarged, base bent outward, tip bent ventrally; outer claw shorter, base and tip not bent. Mesocoxa coarsely punctate and granulate; dense patch of long, golden-yellow setae present on lateral face and adjacent sternum. Mesothoracic leg similar to prothoracic leg except mesofemur with elongate patch of long, recumbent golden-yellow setae on posterior surface extending from near base to half femoral length; mesotibia with pair of cleaning fringes nearly ⅔ as long as tibia. Mesotarsal claws dissimilarly shaped, much longer than protarsal claws, laterally flattened, sharply acute; outer claw enlarged, slightly curved, bent about 90° at base then flattened and widened, more than 2× wider than inner claw; inner claw slightly shorter and much narrower. Metacoxa medial surface with longitudinal, sulcate row of coarse, deep punctures; posterolateral surface with dense patch of long, golden-yellow setae. Metathoracic leg similar to other legs except tibia much longer than femur; single cleaning fringe on posterior face about ⅔ as long as tibia; both claws slightly flattened but basically unmodified, stout, shorter than pro- and mesotarsal claws. *Venter.* Hypomeron with large, coarse, closely spaced punctures, more than 2× diameter of lateral pronotal punctures; ventral margin with broadly rounded lobe directed toward coxa; longitudinal band of golden-yellow plastron present on central ventral margin. Prosternum anterior margin raised, bearing two small, ventrally directed spines; distance between spines narrower than labrum; anterolateral margin behind each eye having a small, nearly semicircular notch; prosternal process about 2× as long as wide, with elevated margin; prosternal disc covered with pale, microreticulate plastron, scattered broad, flat, yellow setae, and large circular punctures spaced slightly less than a puncture diameter apart; golden-yellow plastron present laterally. Mesoventrite depressed between mesocoxae; punctation similar to that of prosternum; disc with pale, microreticulate plastron, mesepimeron with band of dense, golden-yellow plastron. Metaventrite depressed between mesocoxae; discrimen sulcate; posteromedial margin with two low, obtuse, ventrally directed processes; punctures more oval than circular in shape, closer together near midline; disc with pale yellow plastron except along midline, most dense laterad and on metepisternum. Abdomen with pale yellow plastron on all surfaces except for areas of bare, shiny cuticle at midline; ventrites 1–4 non-setose, ventrite 5 with fine, scattered setae; punctures not as large as those on thoracic sternites, becoming progressively smaller with each succeeding ventrite; punctures evenly spaced but less dense than on thoracic sternites; ventrite 1 anterior margin between metacoxae smoothly arcuate; ventrites 1–4 moderately convex at lateral ¼; ventrite 5 with two basolateral swellings each bordered by a shallow depression, apical ⅓ depressed and margin broadly rounded. *Genitalia* (Fig. 2). Elongate, narrow. Phallobase as long as parameres, narrowest at basal ⅓. Paramere in dorsal and ventral views (Fig. 2a, c) sinuous, moderately narrow, narrowest ⅔ distance from base; apex bluntly rounded, clasping tip of penis; paramere in lateral view (Fig. 2b) mostly parallel-sided, apical 1/5 expanded, paddle-like, tip curved slightly ventrad; inner surface shallowly canaliculate. Penis barely shorter than parameres, thin; base about as wide as paramere base, gradually narrowed to just past midlength then widening slightly; apex pointed; corona and fibula absent; basal apophyses about 1/4 as long as phallobase.

**Allotype female.** Length (excluding head), 4.25 mm; width, 2.00 mm. Pronotum 1.25 mm long, 1.55 mm wide; elytron 3.15 mm long, 1.00 mm wide. Secondarily sexually dimorphic as follows: pronotum with two, moderately large, oval perforations of the cuticle on either side of midline; all claws normal, not modified, shorter than those of males; anterior margin of prosternum without paired spines; posterior margin of metaventrite without paired, ventrally directed processes. Otherwise, similar to the male.

#### Variation.

**Figure 3.**
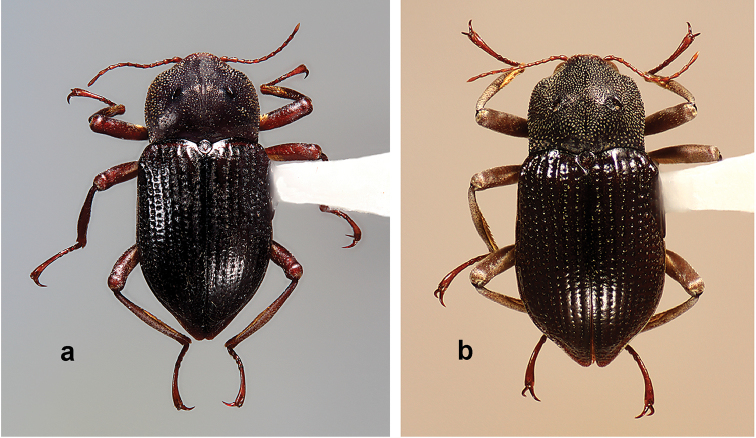
*Amazonopsistheranyi* sp. n. females, dorsal habitus **a** Paratype from Peru, length 4.00 mm **b** Non-paratype from French Guiana, length 4.15 mm.

**Figure 4.**
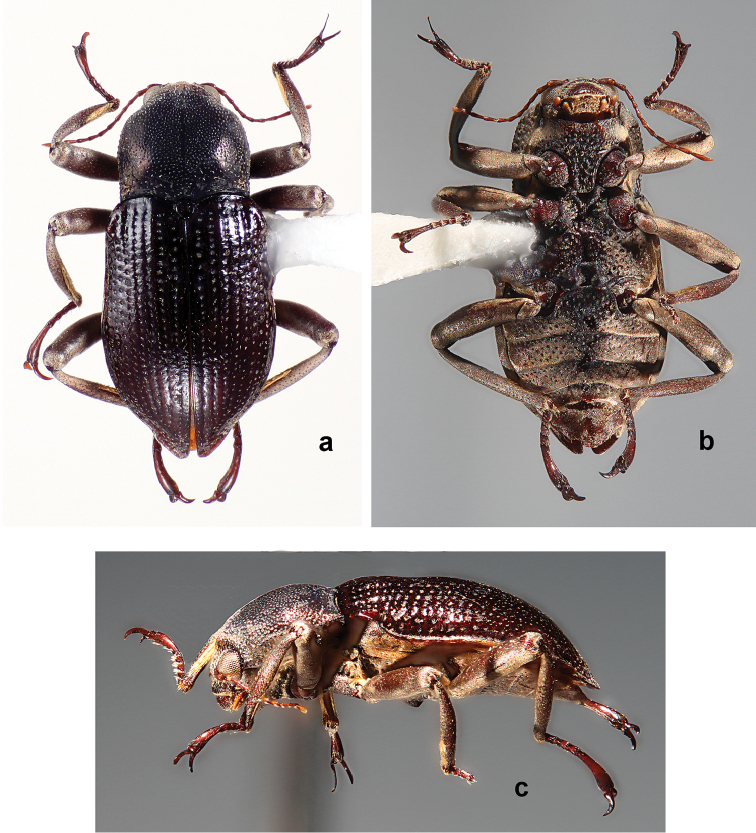
*Amazonopsistheranyi* sp. n., paratype male from Venezuela, length 4.05 mm **a** Dorsal habitus **b** Ventral habitus **c** Lateral habitus.

The most striking variation is the strong secondary sexual dimorphism exhibited by males and females. Males (Figs 1, 4) have the following characteristics which females (Fig. 3) lack: curved, flattened and enlarged pro- and mesotarsal claws; pair of small spines on the prosternal anterior margin; pair of prominent processes on metasternal posterior margin. Females have a pair of oval perforations in the pronotal cuticle. Males and females are of similar size. The males (*n* = 3), 4.05–4.20 mm, and females (*n* = 4), 4.00–4.25 mm, also vary little in length. All specimens showed variability in the amount of plastron present on the elytra. Most individuals have the plastron lacking to some extent on the elytral intervals, with remnants mostly restricted to the lateral margins, striae, and punctures. This is presumably due to abrasion from the environment.

**Figure 5.**
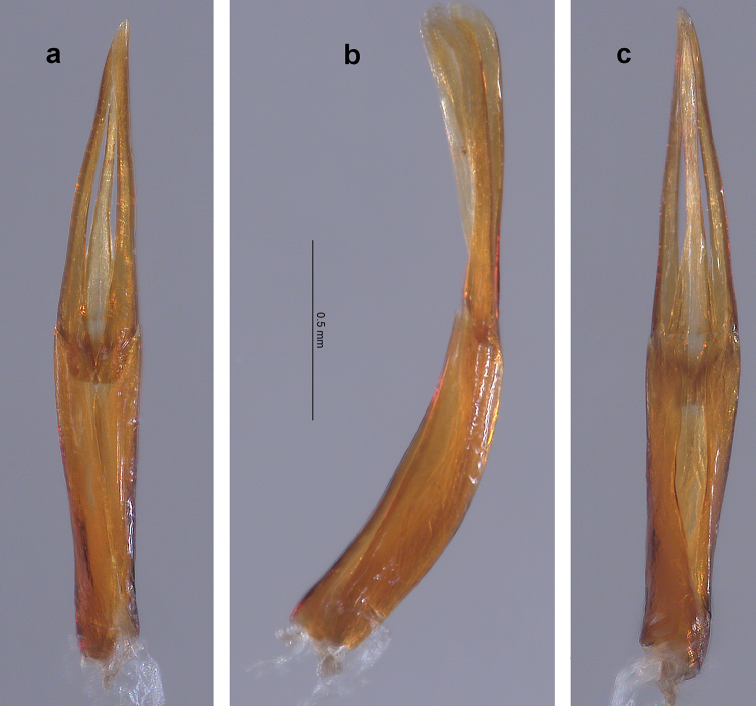
*Amazonopsistheranyi* sp. n. , male genitalia of paratype from Venezuela **a** Dorsal view **b** Lateral view **c** Ventral view.

The single male specimen from Venezuela (Figs 4, 5) varied somewhat from the two males from Peru (Figs 1, 2) in the following manner: body slightly narrower; metaventral processes larger; genitalia (Fig. 5), in dorsal view, with penis thinner in apical ½ and with parameres thinner and not sinuate laterally. The differences are not marked, and with just one individual to compare with only two others, proposing it as a separate species of *Amazonopsis* seems inadvisable.

#### Etymology.

Named for Therany Gonzales Ojeda of ACEER, Puerto Maldonado, Madre de Dios, Peru, the collector of the type series.

#### Distribution.

**Figure 6.**
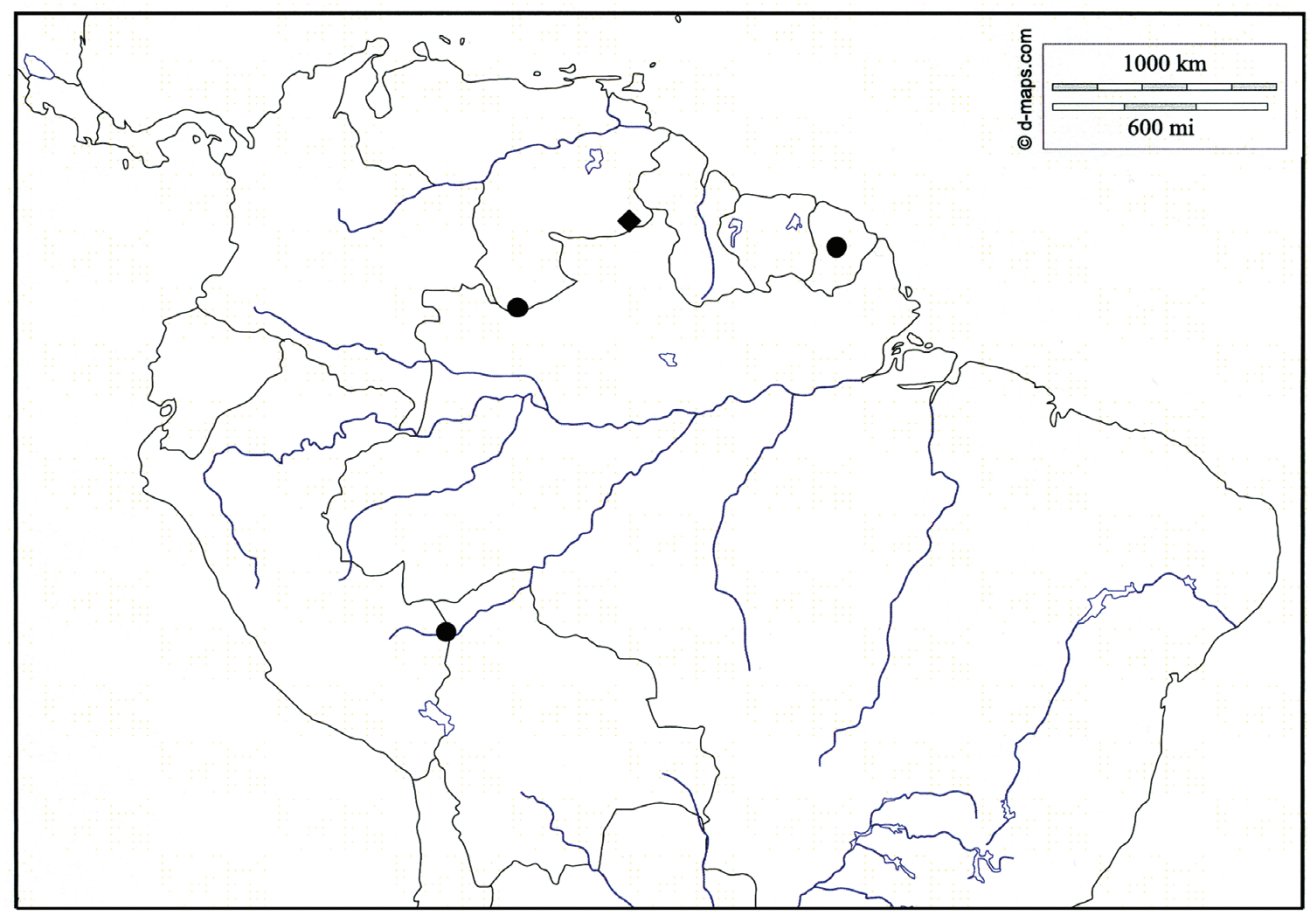
Map of northern South America showing collection localities of *Amazonopsistheranyi* sp. n. (circles) and *A.camachoi* sp. n. (diamond) (map template provided by d-maps of Trets, France).

This species is currently known only from widely separated, single localities in southeastern Peru, southwestern Venezuela, and French Guiana (Fig. 6).

#### Habitat.

**Figures 7, 8.**
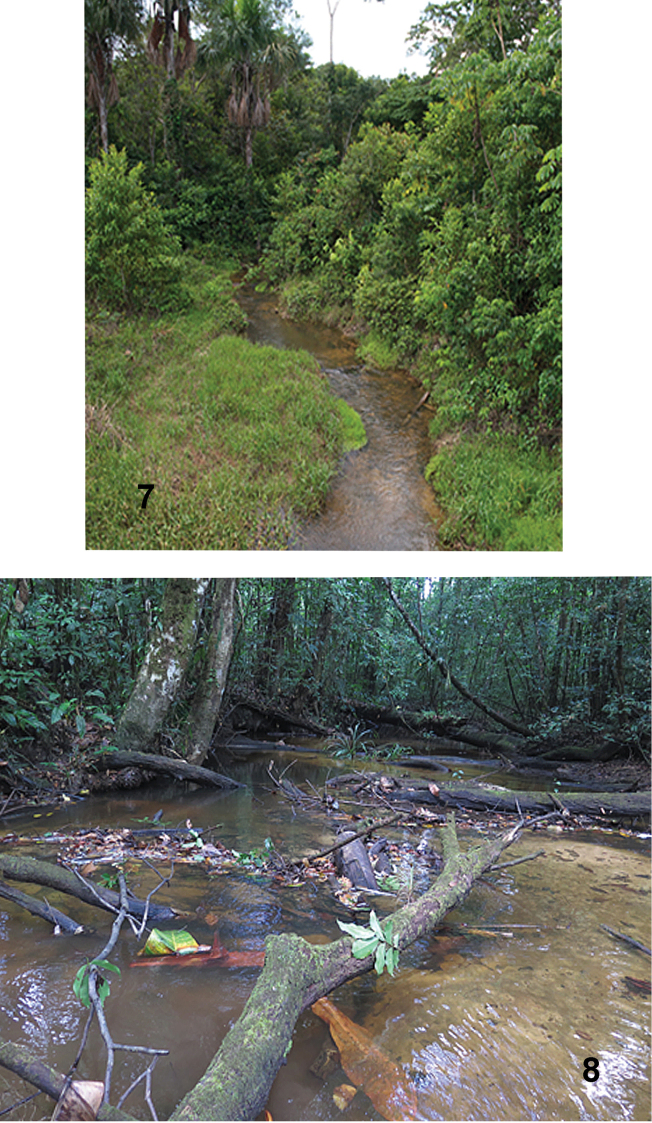
Stream habitats of *Amazonopsistheranyi* sp. n. **7** Quebrada Santo Rosario, Peru, type locality **8** Crique Nouvelle France, French Guiana.

Quebrada Santo Rosario (Fig. 7), the type locality, is a small, second order tributary of the Río Tambopata which feeds the Río Madre de Dios, a major tributary of the Amazon River in Peru. The stream has a small watershed and flows from mostly undisturbed lowland forest at an elevation of 230 m. It was selected as a “control” site by the ACEER project because it was considered non-impacted by human activity. ACEER coordinator Therany Gonzales (in litt.) observed “It is a very clean stream because their [sic] head waters are into a primary forest between the road and the Inambari river that steel [sic] remains pretty well conserved… Collecting macroinvertebrates in this stream was very productive. We got a great diversity and abundance of macroinvertebrates.” The specimens of *Amazonopsis* were collected from artificial leaf pack samples containing the leaves of *Ingaedulis*, a leguminous tree common near streams in the region. Water quality data taken during the ACEER project are as follows: water temperature 25.2 °C, pH 5.13, dissolved oxygen 6.49 mg/L at 25 °C (79%), conductivity 5µS/cm, and turbidity 4 NTU (Sweeney 2014). Observations from my field notes at the site on September 14 and 15, 2013, during which Bill Shepard and I were unsuccessful in collecting additional specimens of *Amazonopsis*, were: water tannin-stained, current slow; bottom substrate of clay and mud with some cobbles; waterlogged wood, a few leaf packs, and root masses present. Quebrada Santo Rosario is intersected by the Interoceanic Highway near the San Juan community about 73 km southwest of Puerto Maldonado.

Crique Nouvelle France (Fig. 8) is a jungle stream in the watershed of the Maroni River, contained wholly within protected Parc Amazonien de Guyane near the community of Saül, in a remote area of central French Guiana. The stream is shallow and sand-bottomed with occasional areas of boulders which form small cascades. Logs, branches and leaf packs litter the channel, and the water is tannin-stained. The specimens were collected from leaf packs or other woody debris. Elevations at the two collection sites are approximately 150 m and 210 m. Water quality measurements taken at the time are as follows: water temperature 23.8 °C, pH 6.6, dissolved oxygen 7.05 mg/L (85.4%), conductivity 44.3 µS/cm, and turbidity 3.61 NTU (Clavier, in litt.).

The information for the Cerro de la Neblina site, a tributary Río Baria, was provided by collector Warren Steiner (in litt.) from his field notes: “small whitewater stream [where I] spent an hour collecting … in leafy side pools near flowing part of stream … ”. The label data with the specimens states “small pool full of dead leaves” as well as an elevation of 140 m. In addition, Spangler (1990: 30) gave a description of the site as follows: “The small whitewater tributary … was about 1 m wide and 1 to 2 cm deep and was shaded by a dense canopy” … “a small, marshy, meandering whitewater rivulet with occasional shallow, leafy pools …”.

#### Associated taxa.

Aquatic byrrhoid beetles collected at the same localities as *Amazonopsistheranyi* include: PERU: *Gyrelmisbrunnea* Hinton, 1940, *G.longipes* Hinton, 1940, *G.maculata* Hinton, 1940, *Hintonelmis* Spangler, 1966 , *Neoelmis* Musgrave, 1935, *Pilielmis* Hinton, 1971, *Portelmis* (Elmidae); *Psephenops* Grouvelle, 1898 (Psephenidae). FRENCH GUIANA: *Cylloepus* Erichson, 1847, *Gyrelmisbrunnea*, *G.nubila* Hinton, 1940, *G.spinata* Hinton, 1940, *G.thoracica* Hinton, 1940, *Heterelmis* Sharp, 1882, *Hexacylloepus* Hinton, 1940, *Hintonelmisperfecta* (Grouvelle, 1908), *Macrelmistereus* (Hinton, 1946), *Neoelmis*, *Pilielmisapama* Hinton, 1971, *Phanocerus* Sharp, 1882 (Elmidae); undescribed genus/species (Protelmidae); *Dryops* Oliver, 1791, *Elmoparnuscollinsae* Spangler & Steiner, 1983, *Platyparnusbollowi* (Hinton, 1939), *P.frater* (Hinton, 1939) (Dryopidae); *Lutrochus* Erichson, 1847 (Lutrochidae). VENEZUELA: *Gyrelmis* Hinton, 1940, *Hexacylloepus*, *Neoelmis*, *Pilielmis*, *Stegoelmisfera* Spangler, 1990, *S.geayi* (Grouvelle, 1908), *S.tuberosa* Spangler, 1990 (Elmidae); new genus (Protelmidae); *Dryops*, *Pelonomus* Erichson, 1847 (Dryopidae). Note: Venezuelan records are cited from Spangler (1990: 30).

### Amazonopsis camachoi sp. n.

Taxon classificationAnimaliaColeopteraElmidae

http://zoobank.org/A5C08C02-A65B-45DA-B485-F5341BD18185

Figs 9 , 10

#### Type material.

**Holotype** male deposited in MALUZ, labeled: “VENEZUELA, Bolívar / Muncipio Gran Sabana / El Paují. 25/IV/2004 al / 02/V/2004 J. Camacho, / J. Perozo, Col. // 04°28'06" N / 61°35'38" W / 880msnm // MALUZ 10031 / LUZ-Venezuela // HOLOTYPE / *Amazonopsis* / *camachoi* Barr” [red label, handwritten].

#### Diagnosis.

**Figure 9.**
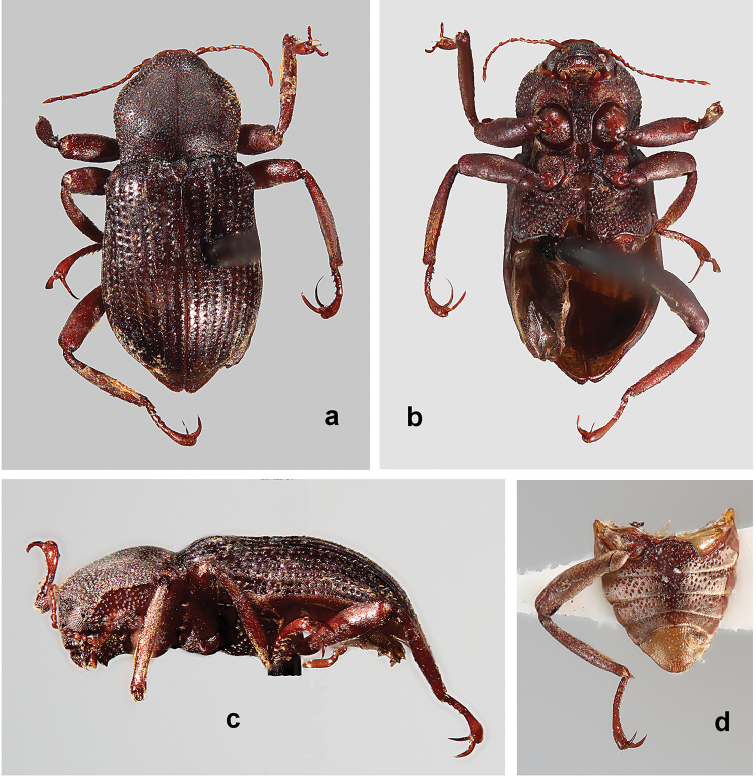
*Amazonopsiscamachoi* sp. n., holotype male, length 4.70 mm **a** Dorsal habitus **b** Ventral habitus, lacking abdomen and right metathoracic leg **c** Lateral habitus, lacking abdomen **d** Ventral habitus of abdomen and right metathoracic leg.

**Figure 10.**
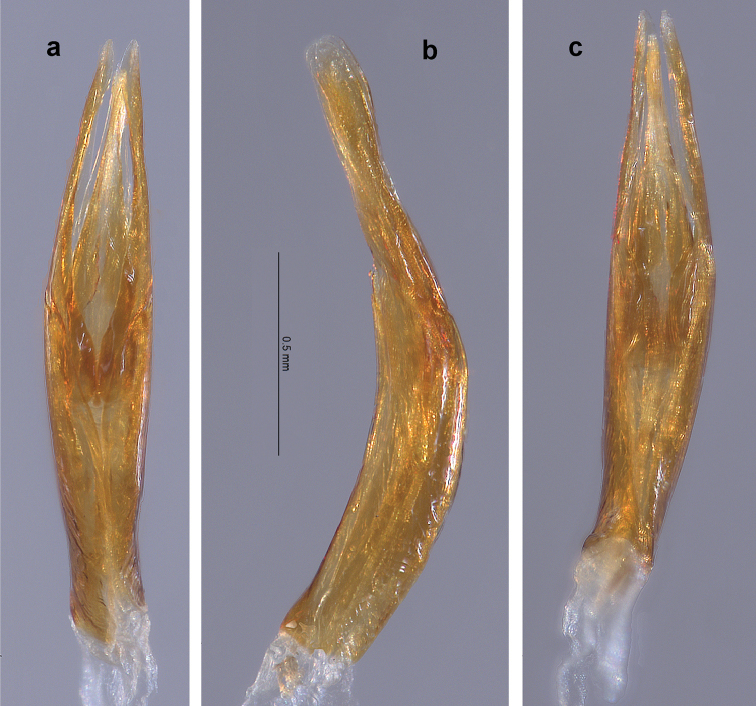
*Amazonopsiscamachoi* sp. n., male genitalia of holotype **a** Dorsal view **b** Lateral view **c** Ventral view.

*Amazonopsiscamachoi* males (Figs 9, 10) differ from those of *A.theranyi* (in parentheses) (Figs 1, 2, 4, 5) by the following characters: elytra with pronounced protuberances at humeral angles (low protuberances), third elytral interval prominently raised (slightly raised), odd-numbered intervals with longitudinal rows of thick setae (all intervals with fine, sparse setae); protarsomere 5 with two apical clusters of long, curved setae extending well beyond base of claws (sparse setae barely extending to base of claws); protarsal claws short, strongly curved (elongate, moderately curved), outer claw with inner tooth (without inner tooth), outer claw width narrower than that of inner claw (width similar); outer mesotarsal claw as long as tarsomere 5 (shorter than tarsomere 5) and at least 3× wider than inner claw (2× wider); distance between prosternal spines as wide as labrum (distance narrower); metaventrite with pair of prominent lobe-like processes (processes less prominent, tooth-like); male genitalia with parameres very narrow and straight (wider and sinuate), penis much wider at base than paramere base (width subequal), phallobase longer than parameres (length subequal).

#### Description.

**Holotype** male (Figs 9, 10). Length, 4.70 mm; width, 2.00 mm. Cuticle mostly covered with pale, thin microreticulate layer dorsally and ventrally, with thicker, pale yellow plastron ventrolaterally on thoracic sterna and abdominal ventrites; cuticle shiny, dark reddish-brown where exposed. *Antenna*. Yellow-brown. Antennomeres 1–10 clavate, antennomere 11 fusiform; antennomeres 1 and 2 each stouter than 3–10 which are of similar size and shape; antennomeres 3–10 each with dense tuft of setae at apicoventral margin, overlapping base of next; antennomere 11 with a faintly visible, elongated patch of short setae near the ventral apex. *Head*. Vertex, frons and clypeus covered with pale, microreticulate plastron and broad, flat, yellow setae. Clypeus dark brown, barely emarginate at center of apical margin, setae slightly less dense than on vertex and frons. Labrum red-brown, barely emarginate, apicolateral angles broadly rounded; surface with small, evenly spaced punctures and short, fine setae; apical and lateral margins with fringe of pale, dense setae, longest laterally. Mandible with three short, rounded, apical teeth. Maxillary palpus yellow-brown; palpomere 4 slightly flattened and curved, longer than 1–3 combined, with oval patch of sensillae at apex. Labial palpus with palpomeres 1 and 2 short, yellow-brown; palpomere 3 longer than 1 and 2 combined, yellow-brown, ovoid and moderately flattened. *Pronotum*. Length, 1.40 mm; width, 1.60 mm. In dorsal view, lateral margins coarsely granulate, evenly arcuate; anterior margin trisinuate, emarginate at middle; anterolateral angles acute, depressed. In lateral view, disc slightly flattened at anterior ⅔. Disc covered with pale microreticulate plastron and closely spaced, coarse punctures; punctures larger and deeper near lateral and apicolateral margins, smaller and shallower, often indistinct, over much of central disc; punctures generally spaced a diameter apart; punctures lined with plastron and associated with very short, erect setae; anteromedial disc and lateral areas with broad, flat, recumbent, yellow setae. Center of midline with narrow, slightly impressed, mostly bare, longitudinal line; length about ¾that of pronotum. Scutellum slightly ovate. *Elytron*. Length, 3.30 mm; width, 1.00 mm. Surface covered with pale, thin microreticulate plastron; punctures striate, deep, coarse, lined with plastron; strial intervals 1, 3, 5, 7, 9 each with partial, longitudinal rows of broad, flat, recumbent, yellow setae (originally complete rows, now partly abraded). Humeral angle with prominent, knob-like, posteriorly directed protuberance; base of third interval conspicuously swollen and raised; lateral margins smooth, recurved with narrow, longitudinal band of hypomeron plastron visible; shallow sulcus about one interval wide adjacent to lateral margin, extending from humeral angle to apical 1/5; elytra constricted at apical 1/5 at point of linkage with abdominal ventrite 4 lateral lobe; apex evenly rounded, moderately produced. *Legs*. Femora and tibiae covered by thin layer of dense plastron, sparsely setose and shallowly punctate; tarsus red-brown, without plastron. Procoxa posterior surface coarsely punctate. Prothoracic leg with tibia longer than femur, tarsus shorter (note: description is of right protarsus; left is missing); profemur with oval patch of long, recumbent pale yellow setae on anterior surface near base; protibia with pair of cleaning fringes, anterior fringe nearly ½ tibial length, posterior fringe about 1/4 tibial length; protarsus with tarsomeres 1–4 bearing tufts of long, curved setae in two rows at apicoventral margins; tarsomere 5 longer than the others combined, with moderately long setae on ventral surface and two apical clusters of long, curved, golden-yellow setae which extend well past base of claws. Protarsal claws short, stout, slightly twisted, laterally flattened, dissimilarly shaped; inner claw slightly bent at base, distal ½ widened, tip broadly acute and bent ventrally; outer claw not bent at base, inner tooth present about ⅓ distance from base, distal ½ narrowed, apex narrowly acute. Mesocoxa coarsely punctate and granulate; dense patch of long, pale yellow setae present on lateral face and adjacent sternum. Mesothoracic leg similar to prothoracic leg except mesofemur with patch of long, recumbent setae on basal ⅓ of posterior surface; mesotibial cleaning fringes ⅔ as long as tibia; tarsomere 5 with long, sparse, apical setae. Mesotarsal claws dissimilarly shaped, much longer than protarsal claws, laterally flattened, sharply acute; outer claw enlarged, as long as tarsomere 5, curved outward, bent more than 90° at base then flattened and widened, at least 3× wider than inner claw; inner claw much shorter, straighter and narrower. Metacoxa medial surface with band of coarse, deep punctures; posterolateral surface with dense patch of long, golden-yellow setae. Metathoracic leg similar to other legs except tibia much longer than femur; single cleaning fringe on posterior face about ⅔ as long as tibia; tarsomere 5 with long, sparse, apical setae; both claws slightly flattened but basically unmodified, stout, shorter than pro- and mesotarsal claws. *Venter*. Hypomeron with large, coarse, closely spaced punctures, more than 2× diameter of lateral pronotal punctures; ventral margin with broadly rounded lobe directed toward coxa; narrow, longitudinal band of pale yellow plastron present on ventral margin. Prosternum anterior margin prominently raised, bearing two small, ventrally directed spines; distance between spines as wide as labrum; anterolateral margin behind each eye having a small, nearly semicircular notch with dorsal margin hook-shaped; prosternal process about 2× as long as wide, with elevated margin; prosternal disc covered with large, irregular punctures and microreticulate plastron, lateral margins with dense plastron. Mesoventrite depressed between mesocoxae; punctation similar to that of prosternum; disc with pale, microreticulate plastron, mesepimeron with band of more dense, pale yellow plastron. Metaventrite depressed between mesocoxae; discrimen sulcate; left posteromedial margin with a prominent, ventrally directed, lobe-like process (right process was destroyed by insect pin); punctures circular and mostly spaced less than a diameter apart; disc with pale plastron except along midline, most dense laterad and on metepisternum. Abdomen with pale yellow plastron on all surfaces except for areas of bare, shiny cuticle at midline; ventrites 1–4 mostly without setae, ventrite 5 with fine, scattered setae; punctures large, becoming progressively smaller with each succeeding ventrite; punctures evenly spaced but less dense than on thoracic sternites; ventrite 1 anterior margin between metacoxae sinuate with two shallow sinuses; ventrite 5 with two basolateral swellings each bordered by a shallow depression, apical ⅓ slightly depressed and margin broadly rounded. Abdomen is separated from rest of body and mounted on a card point. *Genitalia* (Fig. 10). Elongate, narrow. Phallobase longer than parameres, narrowest at basal ⅓. Paramere in dorsal and ventral views (Fig. 10a, c) straight, very narrow, narrowest ½ distance from base; apex narrowly rounded, clasping tip of penis; paramere in lateral view (Fig. 10b) with dorsal margin narrowed at midpoint, apex broadly rounded; inner surface shallowly canaliculate. Penis shorter than parameres; base much wider than paramere base; apical ½ narrow, narrowest point ¾ distance from base; apex narrowly rounded; corona and fibula absent; basal apophyses about ⅓ as long as phallobase.

#### Etymology.

Named for Jesús Camacho of La Universidad del Zulia, Maracaibo, Venezuela, who collected the unique type specimen.

#### Distribution.

This species is currently known only from one locality in southeastern Venezuela (Fig. 6).

#### Habitat.

The collector of the specimen recalled that the stream was small, shallow, sandy, and shaded, and that its waters were dark, tannin-stained, and contained decaying leaves (Camacho, in litt.).

#### Associated taxa.

The only other specimen known from this locality, an unidentified species of *Heterelmis* (Elmidae), is in the MALUZ collection (Camacho, in litt.).

## Discussion

As pointed out in the generic diagnosis, *Amazonopsis* is unique in that no other known genera have males with such bizarrely modified claws or females with pronotal perforations. *Amazonopsis* bears similarities to both *Stenhelmoides* and *Pagelmis*, particularly regarding its extensive dorsal plastron and lack of pronotal and elytral carinae, however, the genus is distinguished by the presence of a lateral projection of the fourth abdominal ventrite and lack of distinctive plastron pattern. *Amazonopsis* also shares some morphological characteristics with *Portelmis*. At this time, lacking phylogenetic analysis, it is not possible to ascertain to which other genus or genera *Amazonopsis* is most closely related.

Given the large geographic range indicated by the four known occurrences (Fig. 6), I was not surprised to discover more than one species among the eight specimens. There are perhaps more species represented than the two species described herein, but I have chosen to be conservative for the following reasons. Regarding *A.theranyi*, the differences between the two male specimens from Peru (Figs 1, 2) and the male specimen from Venezuela (Figs 4, 5), while noteworthy, are insufficient to convince me that they are separate species. Without a larger series of specimens to examine it is difficult to determine if these differences are significant or simply normal variation. Conversely, the specimen described as *A.camachoi* is morphologically distinct, and this outweighed my reluctance to describe a new species from a single, damaged individual. That the two specimens from French Guiana are females lends some doubt to their specific diagnosis. Their morphology is very similar to that of the female specimens from Peru so I have assigned them to *A.theranyi* without designating them as paratypes. To try to resolve this question, subsequent, unsuccessful attempts were made to obtain additional specimens from the collection site in French Guiana (Clavier, in litt.).

Secondary sexual dimorphism, which occurs in several elmid genera and species, involves modifications of various surfaces and structures, particularly on the legs and venter (Kodada et al. 2016). While there are other examples of sexual variation of claw shape (e.g., some *Macronychus* Müller, 1806) (Kodada et al. 2016), none are as radical as the bent and flattened pro- and mesotarsal claws of male *Amazonopsis*. The deep pronotal perforations of female *A.theranyi* are enigmatic and unique among the Elmidae, and like the strange claws of the male, their function is presently unknown. Hypothetically, these pits could be used for chemical communication with males or have a sensory role. Their actual role perhaps could be discovered through destructive morphological examination and SEM photography, however, with precious few specimens in hand at present, that will need to wait for a future opportunity.

The *A.theranyi* specimens examined for this paper are from closed-canopy streams in humid, tropical lowland forests and share habitat similarities (Figs 7, 8). The three streams, with elevations ranging from 140–230 m, are all small, shallow, low-order streams with abundant woody debris and leaf packs; two of the streams are also tannin-stained. The type locality of *A.camachoi* is at the considerably higher elevation of 880 m, but the stream was otherwise similar: shaded, small, shallow, sandy, tannin-stained, and with decaying leaves. The limited data suggest that leaf packs are the primary microhabitat of both species. At the time the specimens were collected, three of the four streams were considered pristine and the remaining one was only slightly impacted by human activities.

At first glance it may seem that the genus *Amazonopsis* displays an unusual geographic distribution pattern (Fig. 6). The site in Peru is located in the Amazon Basin, the two sites in Venezuela are in the Amazon drainage and also the Guiana Shield, and the French Guiana site is on the Guiana Shield. De Granville (1992) used the French term “peri-amazonienne,” which translates to “peri-Amazonian,” to describe distributions of plants that encircle, or partially encircle, the central Amazon Basin. Vanzolini (1973) discussed similar distributions of birds and lizards in relation to postulated tropical forest refuge areas during geologic dry periods. Coincidentally or not, the four occurrences of *Amazonopsis* fall within these postulated refuges as well as conform to the peri-Amazonian distribution models. Likewise, the elmids *Cylloepusolenus* Hinton, 1945 (French Guiana, Brazil, Peru), *Pagelmisamazonica* Spangler, 1981 (Suriname, Ecuador), *Stegoelmisgeayi* (Grouvelle, 1908) (French Guiana, Guyana, Venezuela, Ecuador), and *Stenhelmoidesstrictifrons* Grouvelle, 1908 (French Guiana, Guyana, Venezuela, Brazil, Peru) exhibit similar distribution patterns. At present *Amazonopsis* is known from just four localities in Peru, Venezuela and French Guiana, but it likely occurs in adjacent Brazil, Suriname, and Guyana as well. Although fairly widespread geographically, it may be locally uncommon because of strict habitat requirements and/or be uncommonly collected due to the lack of sampling in the specific habitats and leaf pack microhabitats where it occurs.

## Supplementary Material

XML Treatment for Amazonopsis

XML Treatment for Amazonopsis theranyi

XML Treatment for Amazonopsis camachoi
